# COVID-19 vaccines are effective at preventing symptomatic and severe infection among healthcare workers: A clinical review

**DOI:** 10.1016/j.jvacx.2024.100546

**Published:** 2024-08-05

**Authors:** Oliver Galgut, Fiona Ashford, Alexandra Deeks, Andeep Ghataure, Mimia Islam, Tanvir Sambhi, Yiu Wayn Ker, Christopher J.A. Duncan, Thushan I. de Silva, Susan Hopkins, Victoria Hall, Paul Klenerman, Susanna Dunachie, Alex Richter

**Affiliations:** a Institute of Immunology and Immunotherapy, College of Medical and Dental Science, University of Birmingham, Birmingham, UK; b University Hospitals Birmingham NHS Foundation Trust, Birmingham, UK; c NIHR Oxford Biomedical Research Centre, Oxford University Hospitals NHS Foundation Trust, Oxford, UK; d College of Medical and Dental Science, University of Birmingham, Birmingham, UK; e Translational and Clinical Research Institute Immunity and Inflammation Theme, Newcastle University, Newcastle, UK; f Department of Infection and Tropical Medicine, Newcastle Upon Tyne Hospitals NHS Foundation Trust, Newcastle, UK; g Department of Infection, Immunity and Cardiovascular Disease, University of Sheffield, Sheffield, UK; h Vaccines and Immunity Theme, Medical Research Council Unit The Gambia at the London School of Hygiene and Tropical Medicine, PO Box 273, Fajara, the Gambia; i United Kingdom Health Security Agency, Colindale, London, UK; j Faculty of Medicine, Department of Infectious Disease, Imperial College London, London, UK; k NIHR Health Protection Research Unit in Healthcare Associated Infection and Antimicrobial Resistance, University of Oxford, Oxford, UK; l Translational Gastroenterology Unit, University of Oxford, Oxford, UK; m NDM Centre For Global Health Research, Nuffield Department of Clinical Medicine, University of Oxford, Oxford, UK; n Mahidol-Oxford Tropical Medicine Research Unit, Bangkok, Thailand

**Keywords:** SARS-CoV-2, COVID-19, Healthcare worker, Vaccine, mRNA, Adenovirus vector

## Abstract

**Introduction:**

Health care workers (HCWs) have been at increased risk of infection during the SARS-CoV-2 pandemic and as essential workers have been prioritised for vaccination. Due to increased exposure HCW are considered a predictor of what might happen in the general population, particularly working age adults. This study aims to summarise effect of vaccination in this ‘at risk’ cohort.

**Methods:**

Ovid MEDLINE and Embase were searched, and 358 individual articles were identified. Of these 49 met the inclusion criteria for review and 14 were included in a meta-analysis.

**Results:**

Participants included were predominantly female and working age. Median time to infection was 51 days. Reported vaccine effectiveness against infection, symptomatic infection, and infection requiring hospitalisation were between 5 and 100 %, 34 and 100 %, and 65 and 100 % (respectively). No vaccinated HCW deaths were recorded in any study. Pooled estimates of protection against infection, symptomatic infection, and hospitalisation were, respectively, 84.7 % (95 % CI 72.6–91.5 %, *p* < 0.0001), 86.0 % (95 % CI 67.2 %-94.0 %; p < 0.0001), and 96.1 % (95 % CI 90.4 %-98.4 %). Waning protection against infection was reported by four studies, although protection against hospitalisation for severe infection persists for at least 6 months post vaccination.

**Conclusions:**

Vaccination against SARS-CoV2 in HCWs is protective against infection, symptomatic infection, and hospitalisation. Waning protection is reported but this awaits more mature studies to understand durability more clearly. This study is limited by varying non-pharmacological responses to COVID-19 between included studies, a predominantly female and working age population, and limited information on asymptomatic transmission or long COVID protection.

## Introduction

Severe Acute Respiratory Syndrome Coronavirus 2 (SARS-CoV-2) has been responsible for the on-going worldwide pandemic since the virus was first identified in 2019. Rapid development, licensing and delivery of a number of different vaccine type [1] have altered the disease course of coronavirus disease 2019 (COVID-19). Vaccination is highly efficacious in preventing infection and severe disease in phase III trials [2], [3], [4], [5] and has been evidenced by ‘real world’ effectiveness data showing reduced hospitalisation and improved survival, including with several SARS-CoV-2 variants of concern (VOCs) [6], [7], [8], [9]. However, despite huge improvements in outcome, rates of infection have remained high and re-infections are not uncommon. The reasons for this are not fully understood, but include rapid waning of immunity [10], [11], [12], immune escape of new SARS-CoV-2 variants [13], [14], [15], [16], and suboptimal generation of mucosal protective immune responses [17], [18], [19].

An ideal population to study the relationship between SARS-CoV-2 infection and vaccination is healthcare workers (HCWs). HCWs are one of the few groups who been prioritised for vaccination and so evidence of (re)infection from vaccine waning is likely to occur first in this group. In addition, they have had regular access to screening and diagnostics enabling more rigorous estimates of rates SARS-CoV-2 infection. As a result, there have been many single centre and national healthcare worker studies which can be usefully summarised to understand the benefits of vaccination in this ‘at-risk’ cohort.

This systematic review with meta-analysis was conducted to assess the vaccine effectiveness (VE) of a primary vaccine course against COVID-19 infection, symptomatic infection, hospitalisation, and death among healthcare workers. Collating and understanding the relationship between infection and vaccination in a working age population is important to guide policy to maintain the health of this key workforce but also inform risk in the wider working age population.

## Methods

### Search strategy

**Fig. 1:**
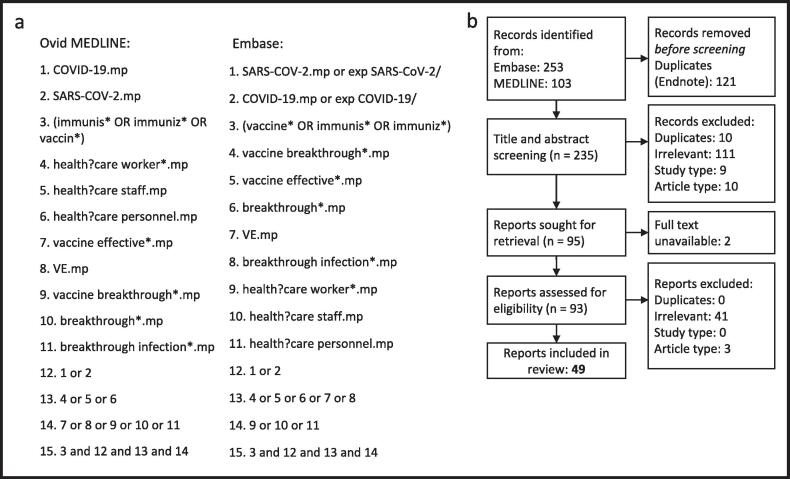
**(a) Search Strategy and (b) PRISMA Diagram.** Panel a indicates the terms used to search Ovid MEDLINE and Embase and their combinations. PRISMA diagram shows number of studies removed at each step in the study selection process. PRISMA=Preferred Reporting In Systematic Reviews and Meta Analyses.

Ovid MEDLINE and Embase were searched on 11th November 2022 using combinations and variations of the terms “COVID-19”, “SARS-CoV-2”, “vaccination”, “immunisation”, “healthcare worker”, “vaccine effectiveness”, and “breakthrough infection” (Fig. 1a). Search terms were chosen based on exploratory searches and the supporting literature review found in Hall *et al*. [20] The papers were extracted and then independently assessed for inclusion by FA and OG, disagreements were resolved by consensus.

This review identified HCW observational studies of COVID-19 VE against infection including the groups any infection (including asymptomatic and symptomatic), symptomatic infection alone, hospitalisation, and death. Studies were included if they compared HCWs that had received a full primary course of vaccination to unvaccinated HCWs. Time from second dose of primary vaccine schedule to infection and risk factors that might predict infection were extracted. Studies were included if the SARS-CoV-2 infection was confirmed by PCR or antibody seroconversion. This review included studies regardless of vaccine methodology but only included mRNA and adenoviral vector vaccines in the meta-analysis. In addition, only studies that define vaccine breakthrough (VBT) as SARS-CoV-2 infection ≥ 14 days after completing a primary vaccination course were eligible for meta-analysis.

No publications were excluded based on country, race, gender, time, SARS-CoV-2 variant, or language. However, articles were only included if full text was available. Any studies that did not present healthcare worker data separately from other workers or pooled data from partially and completely vaccinated people were excluded. Case reports, case series, controlled trials, systematic reviews, and meta*-*analyses were excluded. Editorials, commentaries, interviews, abstract only, and conference papers were excluded. Finally, studies that only assessed immunogenicity of vaccines were excluded. All studies from beginning of the COVID-19 pandemic in 2020 to the date of literature search were eligible.

### Statistical methods

Studies that presented individual participant data were transformed to a hazard ratio (HR) using a complimentary log–log link equation and pooled with reported HRs and incidence rate ratios using a three level random effects model to produce an estimate of HR [21], [22], [23], which was used to calculate an estimate of VE:

$$\mathrm{VE}=\left(1-HR\right)\times 100$$

Sub-group analyses by vaccine type are presented. Studies with empty cells had a treatment arm continuity correction with sum of one prior to transformation with complimentary log–log [24]. Unless otherwise specified, point estimates are presented with 95 % confidence intervals (CI) and medians are presented as medians and interquartile range (IQR).

Estimates of overall proportions are an average of the estimates provided by studies weighted by their population size. Median age and time to infection are calculated using a median of medians approach. Studies presenting mean was converted to a median using techniques described by Wan *et al.* [25]. All analyses were conducted using R (ver. 4.2.2) [26], RStudio [27], and the meta [28] and metafor [29] packages.

## Results

Forty-nine studies of 866,163 HCWs were identified as suitable for inclusion in the review (Fig. 1b). These studies were conducted in Brazil (1) Canada (4), Finland (1), France (1), India (12), Indonesia (2), Iraqi Kurdistan (1), Ireland (1), Israel (2), Italy (8), Japan (1), Portugal (1), Qatar (1), Spain (3), UK (2), and the USA (8) (Table 1). One study [30] required translation from Spanish.

**Table 1:** **Characteristics of included studies.**^†^These periods refer to the time periods studies report as contributing to their calculation of VE, which was not necessarily the length of the while study (e.g Larese-Filon *et al* conducted their study from March 2020 to May 2021, but their VE estimate was calculated from data collected between March 2021 and May 2021. *calculated from data presented in paper. SD=Standard deviation, NR=not reported. Age is presented as median (IQR) unless otherwise stated.

**Reference**	**Country**	**Design**	**Size**	**Follow-up After Vaccination**	**Time Period of Vaccine Assessment^†^**	**% Female**	**Age**
Alishaq *et al*	Qatar	Case Control	328	5 months	December 2020 – May 2021	49 %	39 (33–47)
Allen *et al*	Ireland	Cross-sectional	5,085	4 months	December 2020 – April 2021	78 %	40 (30–49)
Almufty *et al*	Iraqi Kurdistan	Cohort	944	12 months	March 2021 – March 2022	55 %	NR
Anshory *et al*	Indonesia	Cohort	184	6 months	January 2021 – September 2021	57 %	Mean 35.5 (SD±9)
Arriola *et al*	Peru	Cohort	290	3.7 months	February 2021 – May 2021	74 %	45 (38–52)
Basso *et al*	Italy	Cohort	4,394	10 months	February 2021 – November 2021	32 %	Mean 47 (SD±11)
Behera *et al*	India	Case Control	670	2 months	April 2021 – May 2021	45 %	Mean 29.1 (NR)
Bianchi *et al*	Italy	Cohort	6,136	5 months	January 2021 – May 2021	60 %	Mean 45 (SD±13)*
Carazo *et al*	Canada	Case Control	58,476	3.5 months	January 2021 – June 2021	83 %*	40 (30–51)*
Chico-Sanchez *et al*	Spain	Case Control	624	6 months	January 2021 – June 2021	76 %	NR
Consonni *et al*	Italy	Cohort	5,596	16.5 months	December 2020 – May 2022	71 %	36.8 (NR)*
Contractor *et al*	India	Cohort	2,762	10 months	January 2021 – October 2021	45 %	Mean 32.3 (SD±8.3)
Dhumal *et al*	India	Cohort	1,806	Median 3.1 months	January 2021 2020 – June 2021	44 %	32 (Range 18–64)
El Adam *et al*	Canada	Case Control	2,511	9 months	January 2021 – October 2021	87 %	45 (38–54)
Fabiani *et al*	Italy	Cohort	6,423	3 months	December 2020 – March 2021	57 %	Mean 47 (SD±11)
Gaio *et al*	Portugal	Cohort	2,213	11 months	December 2020 – November 2021	81 %	44.3 (36.3–53.5)*
Hall *et al*	UK	Cohort	35,768	10 months	December 2020 – September 2021	84 %	46 (36–54)
Jacobson *et al*	USA	Cohort	660	4.5 months	December 2020 – April 2021	70 %*	Mean 38 (SD±12)*
Kale *et al*	India	Cohort	1,858	4 months	January 2021 – May 2021	NR	NR
Katz *et al*	Israel	Cohort	1,250	3 months	December 2020 – February 2021	80 %	45 (38–57)
Kaur *et al*	India	Cohort	1,500	2 months	NR	32 %	Mean 39 (SD±13)
Kemp *et al*	India	Cohort	5,100	1 month	April 2021	NR	NR
Knobel *et al*	Spain	Cohort	16,723	4.5 months	December 2020 – April 2021	76 %	39 (SD±12)
Lan *et al*	USA	Cohort	4,615	9 months	December 2020 – September 2021	76 %	Mean 45 (SD±13)
Larese Filon *et al*	Italy	Cohort	4,251	3 months	March 2021 – May 2021	69 %	Mean 48 (SD±11)
Malhotra *et al*	India	Cohort	12,237	Median 1. Months	April 2021 – June 2021	34 %	Mean 36 (SD±11)
Marra *et al*	Brazil	Cohort	13,813	6 months	January 2021- August 2021	69 %	Mean 35 (IQR 28–42)
Mendola *et al*	Italy	Cohort	5,605	3 months	February 2021 – May 2021	73 %	45 (34–55)*
Moncunil *et al*	Spain	Cohort	578	12 months	March 2021 – June 2021	73 %	43 (SD±12)
Muhsen *et al*	Israel	Cohort	9,162	3 months	January 2021 – April 2021	80 %	47 (28–66)
Murugesan *et al*	India	Cohort	11,405	3.5 months	April 2021 – May 2021	59 %	33.9 (NR)
North *et al*	USA	Cohort	2,247	2 months	December 2020 – April 2021	78 %	37 (30–50)
Paris *et al*	France	Cohort	8,165	4 months	January 2021 – May 2021	NR	39 (31–49)*
Poukka *et al*	Finland	Cohort	427,905	10 months	December 2020 – October 2021	86 %	46 (35–57)*
Pramod *et al*	India	Case Control	720	4.5 months	March 2021 – May 2021	50 %	34 (28–43)*
Richterman *et al*	USA	Case Control	7,098	4 months	July 2021 – April 2022	81 %	NR
Rifai *et al*	India	Cohort	155	6 months	January 2021 – September 2021	55 %	Range 26–59
Rivelli *et al*	USA	Cohort	12,754	4 months	December 2021 – July 2021	85 %	Mean 42.7 (SD±12.2)
Robilotti *et al*	USA	Cohort	13,658	8 months	December 2020 − August 2021	71 %	Range 22–69
Robilotti *et al*	USA	Cohort	20,857	Median 11.2 months	December 2021 − January 2022	NR	NR
Rovida *et al*	Italy	Cohort	4,066	4 months	January 2021 − April 2021	NR	NR
Soegiarto *et al*	Indonesia	Cohort	2,714	5 months	January 2021 − June 2021	47 %	Mean 36.4 (SD±9.9)
Swift *et al*	USA	Cohort	71,152	Median 2.9 months	January 2021 − March 2021	70 %	41 (NR)
Trunfio *et al*	Italy	Case Control	165	4 months	December 2020 − April 2021	80 %	49 (37–58)
Ujjainya *et al*	India	Cohort	597	3 months	February 2021 − June 2021	NR	39 (Range 22–75)
Vaishya *et al*	India	Case Control	28,342	5 months	January 2021 − June 2021	53 %	Mean 33 (Range 18–80)
Yamamoto *et al*	Japan	Case Control	105	4 months	June 2021 − September 2021	53 %	30 (25–44)
Yassi *et al*	Canada	Cohort	21,248	5 months	December 2020 − May 2021	NR	NR
Yassi *et al*	Canada	Cohort	21,248	10 months	January 2021 − November 2021	NR	NR

The mean follow-up of all included studies was 5.7 months (range 1–16.5), 74 % (range 32–87 %) of participants were women, and the median age was 39 (IQR 35–45) (Table 1). Studies defined VBT as a positive test for SARS-CoV-2 either ≥ 7 days or ≥ 14 days following completion of a primary vaccination course. An infection was most commonly defined by a positive PCR test, although Kale *et al*. [31] and Moncunil *et al.* [32] used PCR or anti-N antibody seroconversion and Poukka *et al.* [33] used “laboratory confirmed” with no further explanation. Included studies covered the period from December 2020 to May 2022, thereby providing information about VE against infection by wild-type, alpha, delta, and omicron BA.1 VOCs.

### Prevention of all infection

Sixteen studies [31], [32], [34], [35], [36], [37], [38], [39], [40], [41], [42], [43], [44], [45], [46], [47], [48] presented time to SARS-CoV-2 infection (symptomatic or asymptomatic). The overall median time to infection was 51 days (IQR 31.5–88) after completing the primary 2-dose vaccination course.

An overall estimate of VE against any SARS-CoV-2 infection was reported by 26 studies [30], [33], [38], [41], [42], [43], [46], [49], [50], [51], [52], [53], [54], [55], [56], [57], [58], [59], [60], [61], [62], [63], [64], [65], [66], [67], [68] and ranged from 5 % to 100 %. The variability in estimates can be attributed primarily to the predominant VOC circulating during the study period. 14 [30], [41], [42], [43], [49], [50], [51], [52], [54], [55], [57], [59], [65], [68] studies examine a predominantly pre-delta period, eight [33], [38], [46], [53], [56], [63], [64], [66] include a delta wave, and four cover [58], [61], [62], [67] a predominantly delta period. Only Richterman *et al* [60] investigated exclusively delta and omicron periods, and they reported the lowest estimate of VE (5 % (CI −69–47 %)) of any study. They also reported VE during the delta wave of 73 % (CI 56–84 %) for mRNA1273 (Moderna, USA) and 75 % (CI −52–87 %) for BNT162b2 (Pfizer, USA). However, during the subsequent omicron wave, this fell to only 5 % (CI −69–47 %) for mRNA1273 and 41 % (CI −2–87 %) for BNT162b2. Similarly, Robilotti *et al* [38] found a relative decrease in mRNA VE during the delta wave compared to the pre-delta period; 75.6 % (CI 68.2–81 %) from 94.5 % (CI 92.9–95.8 %). Lan *et al* [53] also provides a similar estimate (76.5 % CI 40.9–90.6 %) for VE against delta variant. The four studies conducted entirely in the delta wave were all from India and investigated either BBV152 (Baharat Biotech, India) or ChadOx1 nCoV-19 (Serum Institute of India, India) and they provide VE estimates ranging from 29 % to 94 %.

**Fig. 2:**
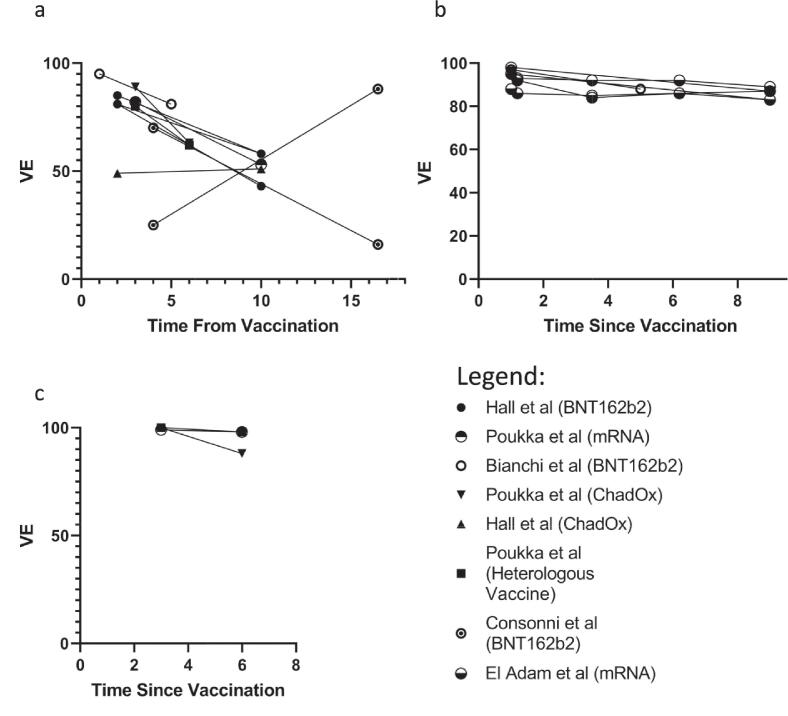
**VE against (a) any SARS-CoV-2 Infection, (b) Symptomatic SARS-CoV-2, and (c) Hospitalisation from SARS-CoV-2 Infection over time.** Time from vaccination is measured in months and calculated from the end of the primary vaccination course. Each estimate is plotted at the end of the period reported, e.g. a study reporting a VE between 3 and 6 months from vaccination will be plotted at 6 months. Studies that feature in more than one panel have the same symbol used throughout. VE=Vaccine Effectiveness, SARS-CoV-2 = Severe Acute Respiratory Distress Syndrome Coronavirus 2.

**Fig. 3:**
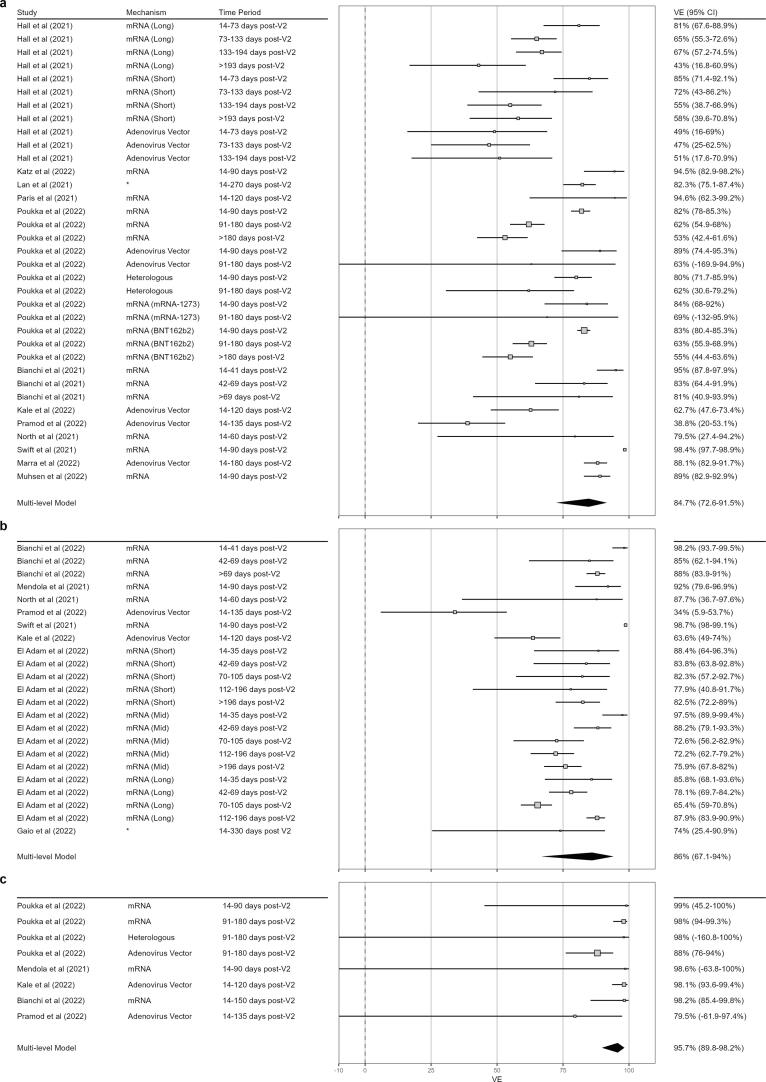
Meta-analysis of VE Estimates against (a) any SARS-CoV-2 Infection, (b) Symptomatic Infection SARS-CoV-2, and (c) Hospitalising Infection SARS-CoV-2. VE estimates of included studies are plotted as a square with horizontal bar representing 95 % CI. A study’s point estimate size is proportional to the weight of that study in the meta-analysis presented. The summary estimate is plotted as a diamond. The time after vaccination, vaccine mechanism assessed, and point estimate with 95 % CI is included. Some studies included estimates for multiple vaccines and/or multiple time points, and these are all presented where included (including different vaccines with the same mechanism where multiple are presented in a paper). Hall *et al*. [69] and El Adam *et al*. [66] present VE for different intervals between V1 and V2; for Hall *et al*. [69] ‘Short’ indicates a dose interval < 6 weeks and ‘Long’ an interval > 6 weeks, for El Adam *et al*. [66] ‘Short’, ‘Mid’, and ‘Long’ indicate dose intervals of 3–5 weeks, 6 weeks, and ≥ 6 weeks, respectively. *Lan *et al*. [53] and Gaio *et al*. [64] pool estimates from mRNA and adenoviral vector vaccines, therefore no mechanism is listed. VE=Vaccine Effectiveness, SARS-CoV-2 = Severe Acute Respiratory Distress Syndrome Coronavirus 2, 95 % CI=95 % Confidence Interval, V1 = first vaccine dose, V2 = second vaccine dose.

Four studies provide estimates of VE across time. Bianchi *et al.* [59], Hall *et al.* [69], and Poukka *et al.* [33] show waning VE, particularly after six months, independent of vaccine type. Consonni *et al.* [70] demonstrate rising VE in HCWs with evidence of SARS-CoV-2 infection prior to vaccination and falling VE in HCWs without a prior history of SARS-CoV-2 infection (Fig. 2).Fig. 3

Only thirteen studies [31], [33], [42], [47], [51], [53], [55], [57], [58], [59], [63], [65], [69] were suitable for meta-analysis estimating VE against any infection. These studies demonstrate a significant VE of 84.7 % (CI 72.6–91.5 %, *p* < 0.0001) despite the relatively short follow up period of these studies (2.5–10 months). The estimated variance components are τ^2^ = 0.83 between studies and τ^2^ = 0.16 within studies. This corresponds to 81.9 % of the total variance being attributable to differences between studies and 15.3 % to differences within studies*.* Sub-group analysis by vaccine methodology showed an improved VE of mRNA over adenovirus vector or heterologous vaccination, although this does not reach statistical significance (Table 2). Studies that performed head-to-head analyses had similar conclusions [33], [69].

**Table 2:** Subgroup analyses of Vaccine Effectiveness against Any Infection and Symptomatic Infection by Vaccine Type. Heterologous refers to two dose courses containing both mRNA and adenoviral vector vaccines. ^a^Lan *et al*[53] removed from subgroup analysis as they pooled multiple vaccine types; ^b^Gaio *et al*[64] removed from subgroup analysis as they pooled multiple vaccine types. VE=Vaccine Effectiveness; 95 % CI=95 % Confidence Interval. All subgroup estimates considered significant at *p* < 0.05.

**Vaccine type**	**VE**	**95 % CI**	**τ^2^**	***p* Subgroup Differences**
**Against any Infection^a^**				**0.051**
mRNA	92.1 %	80.5–96.8 %	2.83	
Adenoviral Vector	70.5 %	42.6–84.9 %	0.53	
Heterologous	73.8 %	51.1–85.9 %	0.14	
**Against Symptomatic Infection^b^**				**<0.001**
mRNA	92.6 %	80.7–97.1 %	1.09	
Adenoviral Vector	51.1 %	12.4–72.7 %	0.15	

The only study that specifically acknowledged the difference between asymptomatic and symptomatic infection was Knobel *et al*. [52] which found mRNA VE against asymptomatic infection to be 90.6 % in the four and a half months following vaccination.

### Symptomatic and severity of infection

Ten studies [31], [49], [50], [51], [58], [59], [62], [64], [66], [71] reported an overall VE against symptomatic SARS-CoV-2, with estimates ranging from 34 % to 100 %. Kale *et al.* [31] and Malhotra *et al*. [62] reported the lowest estimates (34 % CI −16–50 % and 44 % CI 38–50 % respectively). Both studies were conducted in India during a period of delta variant predominance, but, Kale *et al*. [31] assessed ChadOx1 nCoV-19 vaccination and Malhotra *et al.* [62] assessed BBV152. When considering the seven studies [49], [50], [51], [59], [64], [66], [71] that cover a pre-delta period overall estimates of VE ranged from 72 % to 100 %. Bianchi *et al.* [59] and El Adam *et al.* [66] provide estimates of VE over time; Bianchi *et al.* [59] identify waning immunity with BNT162b2 and El Adam *et al.* [66] found consistent protection over time when any mRNA vaccination (maintained when considering a 3- to 5-week, 6 week, or ≥ 7 week dose interval (Fig. 2)).

The pooled VE against symptomatic infection of eight studies [31], [42], [57], [58], [59], [64], [66], [71] was 86.0 % (CI 67.2–94.0 %, *p* < 0.0001). The estimated variance components are τ^2^ = 1.25 between studies and τ^2^ = 0.16 within studies. This corresponds to 85.8 % of the total variance is attributable to between study and 11.0 % to within study heterogeneity*.* Sub-group analysis by vaccine type showed improved VE of mRNA vaccine compared to adenovirus vector or heterologous vaccine strategies (Table 2).

One study compared the time of symptoms between vaccinated and unvaccinated HCWs and found a trend toward shorter illness (median length of illness of 5 days vs 9 days).[37] Symptoms among vaccinated HCWs were typically milder than unvaccinated HCWs [37], [43], [48].

### Hospitalisation and death

Hospitalisation and death were rare events across all included studies – 414 participants were hospitalised [31], [40], [43], [48], [49], [58], [59], [71], [72], [73], [74] and only 10 deaths recorded [31], [38]. All deaths were among unvaccinated HCWs. VE at preventing hospitalisation is estimated to be between 65 % and 100 % [31], [33], [49], [62]. The lowest estimate (65 % CI 46–78 %) came from a study investigating BBV152 in India during the delta wave [62] Poukka *et al.* [33] demonstrate lasting immunity against hospitalising infection (Table 2).

Data suitable for pooling were provided by five studies [31], [33], [58], [59], [71]; with a pooled estimate of VE against hospitalisation of 96.1 % (CI 90.4–98.4 %, *p* < 0.0001). The estimated variance components are τ^2^ = 0 between studies and τ^2^ = 0.69 within studies. This corresponds to 52.2 % of the total variance being attributable to within study heterogeneity. Sparse data prevented a subgroup analysis by vaccine type.

### Risk factors for VBT

Risk factors for VBT were assessed in ten studies [31], [34], [44], [45], [72], [73], [75], [76], [77], [78]. Results were mixed when assessing the effect of age, male gender, and BMI. Basso *et a*l. [76] reported increasing age protective (adjusted Odds Ratio (aOR) 0.95/year 0.94–0.97) and that male gender and BMI>25 increased risk of VBT (aOR 1.57 1.07–2.28 and 1.61 1.08–2.39, respectively). Dhumal *et al.* [45] also found increased risk of VBT in men (aHR 2.06 CI 1.19–3.56) but found that HCWs under 50 years old had no increased risk of VBT (aHR 1.01 CI 0.98–1.04). Alishaq *et al* [75] found age and male gender had no effect (HR 1.02/10 years CI 0.61–1.68 and 1.26 CI 0.51–3.16, respectively), and BMI<30 increased risk of VBT (aOR 2.99 1.2–7.48). Soegiarto *et al.* [77] also found no effect of male gender or age over 40 (aOR 0.887 CI 0.732–1.075 and 0.989 CI 0.808–1.21, respectively); however, they found a trend toward reduced risk in those with BMI a 25–29.9 compared to BMI<25 (aOR 0.81 CI 0.666–1) and no effect of BMI≥30. Almufty *et al.* [73] found no difference in the chance of vaccine breakthrough between men and women (26 % vs 27 %, *p* = 0.817) or for the over- and under-50 s (21 % vs 27 % *p* = 0.119). Anshory *et al.* [78] found no effect of age on breakthrough or female gender (aRR 0.97/year CI 0.93–1.02 and 0.99 CI 0.51–1.96). Rivelli *et al.* [44] found age < 35 increased risk of vaccine breakthrough (aOR 1.76 CI 1.18–2.63). Allen *et al.* [34] found the youngest HCWs (18–29 year olds) to be at highest risk of VBT (aRR 1.3 CI 1.1–1.6 vs 50–59 year olds) and a trend towards increased risk in men compared to women (aRR 1.2 CI 1.0–1.4). Allen *et al.* [34] also assessed the effect of educational attainment and found that decreasing education level increased risk of VBT (secondary education aRR 1.4 CI 1.1–1.8, primary education 1.6 CI 0.9–2.9; both compared to post-graduate education).

Smoking status was found to have no effect on VBT, Alishaq *et al.* [75] reported HR 2.47 (0.69 – 8.90) and Basso *et al.* [76] uOR 0.8 (CI 0.5–1.28). Soegiarto *et al.* [77] reported previous and current smokers as having similar a likelihood of breakthrough as non-smokers (aOR 1.34 CI 0.771–2.331 and aOR 1.032 CI 0.748–1.424, respectively).

Results with specific comorbidities are also mixed. Anshory *et al.* [78] reported that having no comorbidities was not protective (aRR 1.39 CI 0.70–2.75). Rifai *et al.* [72] and Soegiarto *et al.* [77] reported that HCWs with hypertension were more likely to have VBT (38 % vs 20 % and aOR 1.369 CI 1.009–1.859, respectively). In addition, Rifai *et al.* [72] reported increased likelihood of symptomatic infection (64 % vs 24 %) or hospitalising infection (55 % vs 24 %) among hypertensive HCWs. However, Basso *et al.* [76] reported no effect (unadjusted Odds Ratio (uOR) 0.65 CI 0.34–1.23). Basso *et al.* [76] reported diabetes mellitus (DM) increased the risk of vaccine VBT (aOR 2.41 CI 1.04–5.6), while Soegiarto *et al.* [77] reported no effect of DM (aOR 1.269 CI 0.757–2.129). Soegiarto *et al.* [77] also reported no effect cardiovascular or respiratory disease (aOR 0.596 CI 0.253–1.402 and aOR 0.89 CI 0.634–1.251 respectively).

The effect of job role was variable. Basso *et al.* [76] found that VBTs were more frequent among nurse aids and auxiliary personnel (39 %), followed by nurses (36 %) and physicians (14 %). Technicians (7 %) and administrative and support staff (5 %) were less likely to have VBT. Their logistic regression identified only administrative and support staff and technicians to be at decreased risk of VBT when compared to physicians (OR 0.34 CI 0.14–0.70 and 0.32 CI 0.14–0.84, respectively); this was only maintained for technicians in the multivariate analysis (aOR 0.29 CI 0.13–0.67). Likewise Allen *et al.* [34] found that nurses and healthcare assistants had highest risk of VBTs (1.4 CI 1.0–1.8 and 1.8 CI 1.3–2.3; compared to administrative staff). Alishaq *et al.* [75] found that compared to nurses and midwives, clinical support services, administrators, and physicians were at increased risk of VBT (HR 9.15 (CI 1.32–63.64), 4.10 (CI 1.13––14.90), 6.27 (CI 1.20–32.82)). Allied Health Professionals and Non-clinical Support Services appeared to be at similar or slightly increased risk to nurses and midwives (HR 2.99 (CI 0.94 – 9.50) and 4.95 (CI 1.00 – 23.45)). However, Anshory *et al.* [78] found neither resident doctors nor nurses were at higher risk of vaccine breakthrough than specialist doctors (aRR 5.01 CI 0.76–32.94 and aRR 3.15 CI 0.70–14.14, respectively). Almufty *et al.* [73] identified dentists to have highest chance of VBT (43 %), followed by pharmacists and physicians (35 % and 33 % p < 0.001); paramedics had the lowest chance of VBT (8 % p < 0.001), although they had the highest chance of breakthrough requiring ventilation (33 % vs between 1–10 %, *p* < 0.05). Kale *et al.* [31] found patient-facing roles (doctors 22 % and nurses 24 %) had increased risk of infection compared to non-patient facing roles (technicians 7 %, non-medical 4 %, and housekeeping staff 3 % *p* < 0.001). Likewise, Rivelli *et al.* [44] found that staff in clinical roles were at increased risk of breakthrough when compared to non-clinical roles (aOR 2.29 CI 1.36–3.84) and this risk was increased when comparing COVID clinical roles to non-clinical ones (aOR 7.36 CI 2.99–18.09). Likewise, Allen *et al*[34] found that, when compared to HCWs with no patient contact, both HCWs with contact to patients without COVID-19 and HCWs with contact to patients with COVID-19 had increased risk of VBT (aRR 1.3 CI 1.1–1.5 and aRR 1.4 CI 1.1–1.7, respectively). Alsihaq *et al*[75] identified that HCWs with VBT were more likely to reported contact with a COVID-19 case than those without, particularly their spouse (27 % vs 6 % *p* < 0.00001), other close family member (10 % vs 4 % *p* < 0.017), or patient (14 % vs 3 % *p* < 0.00001). Uninfected were more likely to deny contact with individuals with COVID-19 (80 % vs 44 % *p* < 0.0001). However, Almufty *et al*[73] reported no difference in chance of VBT between those working in COVID centres and other healthcare locations (30 % vs 27 %, *p* = 0.551). Anshory *et al*[78] identify contact with COVID-19 as increasing risk of breakthrough when outside the home or hospital (aRR 6.82 CI 1.97–47.98). They assess the effect of Personal Protective Equipment (PPE) use on infection risk. Although the amount of PPE worn had no effect, N95 and KN95 masks were more protective than surgical masks (aRR 0.05 CI 0.01–0.45 and 0.06 CI 0.01–0.51, respectively). Likewise, reporting never wearing a mask increased risk of breakthrough (aRR 7.12 CI 1.88–26.96) [78].

Five studies[44], [45], [70], [74], [79] directly assessed the effect of SARS-CoV-2 infection prior to vaccination. Of these, protective effects were found by Dhumal *et al*[45], Contractor *et al*[79], and Robilotti *et al*[74] (aHR 0.287 CI 0.089–0.917, aHR 0.12 CI 0.07–0.20, IRR 0.635 CI 0.545–0.733, respectively). Consonni *et al*[70] found improved VE between 4- and 16-months post vaccination in those with prior SARS-CoV-2 infection and waning immunity in those without; VE 88 % CI 66–96 % vs 16 % CI 0–43 %. However, Rivelli *et al*[44] found that SARS-CoV-2 infection prior to infection increased likelihood of vaccine breakthrough (uOR 3.18 CI 2.12–4.78). Interestingly, when the effect of other covariates is stratified by pre-vaccination SARS-CoV-2 infection, Rivelli *et al*[44] found that prior SARS-CoV-2 infection has a protective effect.

## Discussion

We performed a clinical review and meta-analysis of observational studies of vaccine effectiveness against SARS-CoV-2 infection among HCWs. Reviewing observational studies provides essential data of the real-world effectiveness of COVID-19 vaccines and by studying HCWs we have limited the influence of social distancing measures on VE estimates. Vaccination is found to be effective against any infection, symptomatic infection, and hospitalisation. Of 866,163 vaccinated HCWs included in this review no individual died of COVID-19. In other similarly age cohorts, small numbers of deaths are recorded, and these are restricted to those over 40s years of age [80], [81], [82]. There was waning protection against infection, particularly past six months, but protection against symptomatic and severe infection persisted. VE reduced with VOCs, particularly omicron BA.1. Sub-group analysis by vaccine type suggests mRNA vaccines are more effective than adenovirus vector vaccines at preventing symptomatic or asymptomatic SARS-CoV-2. In addition, vaccines reduce the severity of disease; vaccinated HCWs were less likely to have symptoms and more likely to have milder symptoms compared to their unvaccinated colleagues. Lowest VE estimates came from India, adenoviral vector or whole inactivated virus vaccines, and VOCs; It is difficult to tease apart the individual effects of these factors as their effects are confounded with each other – particularly the low VE of adenoviral vector and whole inactivated virus vaccines and low VEs reported from India as the studies conducted in India all used adenoviral vector and whole inactivated virus vaccines.

This demonstrates the effectiveness of COVID-19 vaccines in an essential workforce. The waning protection against non-hospitalising infection supports the decision to roll out booster programmes among working age adults at high exposure risk to SARS-CoV-2. This correlates with waning antibody titres seen in similar populations [10], [11], [12], [83], [84], [85] and the longer lasting protection against hospitalising infection suggests an important cellular component to immunity against severe SARS-CoV-2 infection.

Our mixed findings on the effect of risk factors on VE may be explained by the restricted population included. Observational studies of the general population indicate increasing risk with increasing age [81], [86], Hippisley-Cox *et al* [82] found an exponential increase in risk beyond middle age; our inconclusive findings are largely due to all included participants being of a low risk age for breakthrough and the possibility that older HCWs being deployed away from high exposure areas. Hippisley-Cox *et al* [82] reported the risk of vaccine breakthrough being highest at the extremes of BMI, and highest at a BMI of 15 [82]; which may explain the conflicting results of Basso *et al* [76] and Alishaq *et al* [75], who reported BMI>25 and BMI<30 increase risk of VBT (respectively). In the general population, male gender appears to increase risk of VBT and it is difficult to explain the mixed results seen in this review; but it may be due to the gender skew of the studies assessing this (Table 1).

Likewise, the inconsistent effect of profession may be because professional background (e.g. physician or radiographer) does not reliably predict SARS-CoV-2 exposure. Direct contact with a confirmed COVID-19 case increased vaccine breakthrough risk in HCWs and this is similar to the finding that increased background SARS-CoV-2 prevalence increases risk of VBT. [82] The evidence provided by Anshory *et al* [78] supports that the quality and availability of PPE, not professional background, is more directly linked to vaccine breakthrough risk. In addition, job role is confounded by socioeconomic status and education level, both have been linked to increased likelihood of SARS-CoV-2 infection [34], [87], [88].

## Limitations

We acknowledge several limitations to this systematic review. We were unable to account for differences between studies in non-pharmacological interventions at work or home (e.g. lockdowns or social distancing) and PPE quality and availability. We are also unable to account for the effect of variable testing regimens. This is particularly important when assessing protection against asymptomatic infection, where studies with more proactive testing regimens are more likely to detect infection (particularly mild or asymptomatic infection). Recent modelling work suggests that in observational studies testing infrequently or only symptomatic participants, there would be a tendency to underestimate VE [89]. The most common testing regimen of included studies included in our review was regular nasopharyngeal PCR testing or monitoring for seroconversion, which are least vulnerable to this effect [89]. Together, these limitations may explain (in part) the high heterogeneity of the meta-analysis estimates and wide variation in reported VE estimates.

Our comparison of VE between different vaccine types and VOCs is limited by a relative lack of studies conducting direct comparisons. In addition, the cohorts reviewed were typically working age and female – which makes the study results less transferable to other ‘at-risk’ populations. In addition, we may be vulnerable to the healthy worker effect, which can be more pronounced in women [90].

The included literature did not assess vaccination’s effect on nosocomial infection or asymptomatic transmission of SARS-CoV-2, but a systematic review of observational studies suggests that vaccination can reduce onward SARS-CoV-2 transmission (VE against transmission 16–95 %), however this is lower with more recent variants and they could not assess the VE of vaccines targeted to more recent VOCs [91]. In addition, none of the studies included in this review reported VE against long COVID, therefore we could not assess this; however, other work suggests that risk and severity of long COVID is increased by severity of infection [92], [93], [94], [95], [96] and our finding of reduced severity of infection in vaccinated HCWs suggests that, by extension, vaccination may lessen the burden of long COVID among HCWs (a finding seen in other populations [97]). We were unable to assess the effect of race and ethnicity on risk VBT due to the variability of racial and ethnic groupings between countries and limited reported evidence.

## Conclusion

This review demonstrates real world effectiveness of COVID-19 vaccines among a working age population, particularly against severe illness. We find evidence of waning VE against any SARS-CoV-2 infection within 6 months and reduced effectiveness against the delta and omicron VOCs, supporting the decision to provide further booster vaccinations for highly exposed workforces (including HCWs) and ongoing development of new vaccine candidates. Understanding the effectiveness of vaccines in the working age population is useful going forward in terms of maintaining workforce health and economic productivity, and this allows us to focus resource and advice. Further work is needed to identify risk factors for VBT and protection against long COVID.

**Funding**

This work was supported by the United Kingdom Research and Innovation Medical Research Council (MRC) (grant number MR/W02067X/1) and the Huo Family Foundation. CJAD is an MRC Clinician Scientist Fellow (MR/X001598/1). TldS is supported by a Wellcome Trust Intermediate Clinical Fellowship (110058/Z/15/Z). SD is supported by an NIHR Global Research Professorship (NIHR300791). PK is an NIHR Senior Investigator and is funded by the Wellcome Trust (WT109965MA) and National Institutes of Health grant (U19 AI082630).

## CRediT authorship contribution statement

**Oliver Galgut:** Conceptualization, Data curation, Formal analysis, Investigation, Methodology, Project administration, Validation, Visualization, Writing – original draft. **Fiona Ashford:** Data curation, Investigation, Validation, Writing – review & editing. **Alexandra Deeks:** Investigation, Writing – review & editing. **Andeep Ghataure:** Investigation, Writing – review & editing. **Mimia Islam:** Investigation, Writing – review & editing. **Tanvir Sambhi:** Investigation, Writing – review & editing. **Yiu Wayn Ker:** Investigation, Writing – review & editing. **Christopher J. A. Duncan:** Funding acquisition, Writing – review & editing. **Thushan I. de Silva:** . **Susan Hopkins:** Funding acquisition, Writing – review & editing. **Victoria Hall:** Funding acquisition, Writing – review & editing. **Paul Klenerman:** Funding acquisition, Writing – review & editing. **Susanna Dunachie:** Funding acquisition, Supervision, Writing – review & editing. **Alex Richter:** Conceptualization, Funding acquisition, Supervision, Writing – original draft.

## Declaration of competing interest

The authors declare that they have no known competing financial interests or personal relationships that could have appeared to influence the work reported in this paper.

## Data Availability

I have attached code for running meta analysis
